# A Short-Type Peptidoglycan Recognition Protein 1 (PGRP1) Is Involved in the Immune Response in Asian Corn Borer, *Ostrinia furnacalis* (Guenée)

**DOI:** 10.3390/ijms22158198

**Published:** 2021-07-30

**Authors:** Dongxu Shen, Jiayue Ji, Shasha Zhang, Jiahui Liu, Chunju An

**Affiliations:** 1 Department of Entomology, College of Plant Protection, China Agricultural University, Beijing 100193, China; shendongxu0311@163.com (D.S.); jijiayue@cau.edu.cn (J.J.); zhangshasha@cau.edu.cn (S.Z.); jiahuiL95@163.com (J.L.); 2 Jiangsu Key Laboratory of Sericultural Biology and Biotechnology, School of Biotechnology, Jiangsu University of Science and Technology, Zhenjiang 212018, China; 3 Key Laboratory of Silkworm and Mulberry Genetic Improvement, Ministry of Agriculture and Rural Affairs, Sericultural Research Institute, Chinese Academy of Agricultural Sciences, Zhenjiang 212018, China

**Keywords:** agglutination, binding, innate immune response, *Ostrinia furnacalis* (Guenée), peptidoglycan recognition proteins (PGRPs), prophenoloxidase stimulation

## Abstract

The insect immune response is initiated by the recognition of invading microorganisms. Peptidoglycan recognition proteins (PGRPs) function primarily as pattern recognition receptors by specifically binding to peptidoglycans expressed on microbial surfaces. We cloned a full-length cDNA for a PGRP from the Asian corn borer *Ostrinia furnacalis* (Guenée) and designated it as PGRP1. *PGRP1* mRNA was mainly detected in the fat bodies and hemocytes. Its transcript levels increased significantly upon bacterial and fungal challenges. Purified recombinant PGRP1 exhibited binding activity to the gram-positive *Micrococcus luteus*, gram-negative *Escherichia coli*, entomopathogenic fungi *Beauveria bassiana*, and yeast *Pichia pastoris.* The binding further induced their agglutination. Additionally, PGRP1 preferred to bind to Lys-type peptidoglycans rather than DAP-type peptidoglycans. The addition of recombinant PGRP1 to *O. furnacalis* plasma resulted in a significant increase in phenoloxidase activity. The injection of recombinant PGRP1 into larvae led to a significantly increased expression of several antimicrobial peptide genes. Taken together, our results suggest that *O. furnacalis* PGRP1 potentially recognizes the invading microbes and is involved in the immune response in *O. furnacalis*.

## 1. Introduction

Insects lack the acquired immune system possessed by vertebrates and mainly rely on a rapid and effective innate immune system to defend against microbial infections [1,2]. Insect innate immunity consists of a hemolymph-mediated humoral response and a hemocyte-mediated cellular response. Both responses are initiated by the recognition of the invading microbes by insect pattern recognition receptors (PRRs) [3]. PRRs perform surveillance functions by binding to peptidoglycans, polysaccharides, glycoproteins, and glycolipids that are common to groups of microorganisms [4,5]. A variety of proteins, including peptidoglycan recognition protein (PGRP), β-1,3-glucanase-related protein (βGRP), C-type lectin (CTL), galectin, hemolin, hemocytin, scavenger receptor, thioester protein, Draper, and Eater were reported to function as PRRs in insects [6,7,8].

Among these, PGRPs are immunity-related molecules conserved from insects to humans [9,10]. PGRPs are characterized by possessing a highly conserved PGRP domain that is similar to *N*-acetylmuramyl-alanine amidase, such as bacterial amidase and bacteriophage T7 lysozymes [11]. The PGRP domain adopts similar overall folding to that of T7 lysozymes, which contains three peripheral α-helices and a central β-sheet composed of five β-strands [12]. The first PGRP was identified and purified from the hemolymph of the silkworm, *Bombyx mori* [13], and since then, more orthologs have been identified in other insects. For example, *Drosophila melanogaster* has 13 PGRP genes encoding 17 proteins by alternative splicing [14]; *Anopheles gambiae* has 7 PGRP genes encoding 9 proteins [15]; and *B. mori*, *Manduca sexta*, and *Tribolium castaneum* have 12, 14, and 7 PGRPs [16,17,18], respectively.

Insect PGRPs are categorized into two groups based on the protein size: short PGRPs (PGRP-S) and long PGRPs (PGRP-L) [14]. The short PGRPs, with an apparent molecular weight of approximately 20 kDa, have signal peptides and are all extracellular proteins, while the long PGRPs of up to 90 kDa can be intracellular, transmembrane, or extracellular [19]. The expression of most PGRP-Ss is induced in response to bacterial infection, whereas many PGRP-Ls are constitutively expressed [14]. PGRP family members perform diverse functions in the insect innate immune response. Some PGRPs perform amidase activities by hydrolyzing the bond between the *N*-acetylmuramyl group in the glycan strand and the L-alanine in the stem peptide of peptidoglycan and generating non-immunogenic peptidoglycan fragments [11]. These PGRPs are hence classified as catalytic PGRPs and act as immune modulators. For example, *D. melanogaster* PGRP-LB, -SC1a, -SC1b, and SC2 cleave *meso*-diaminopimelate (DAP)-type peptidoglycans from gram-negative bacteria and negatively regulate the Imd pathway [20,21,22]. However, more PGPRs lack amidase activity because of the substitution of key amino acid residues. They only can recognize and bind to, but not cleave, bacterial peptidoglycans [21]. Therefore, they function as sensors for ligand-dependent signaling to activate the prophenoloxidase (PPO) cascade or produce antimicrobial peptides (AMPs) via the Toll or Imd pathway [23,24]. *D. melanogaster* PGRP-SA and PGRP-SD were reported to bind to the Lys-type peptidoglycan from gram-positive bacteria and further trigger the Toll pathway [25]. The silencing of *PxPGRP-S1* expression by RNAi in *Plutella xylostella* (L.) could decrease the expression of antimicrobial peptides in larvae [26]. Overexpression of PGRP-LE in *D. melanogaster* led to the activation of the PPO cascade [27]. Other PGRPs, including *B. mori* PGRP-S4, *M. sexta* PGRP1, and *Tenebrio molitor* PGRP-SA, have also been revealed to work as receptors in the response to bacterial infections [28,29,30]. Furthermore, some PGRPs were even demonstrated to be effectors to kill invading microbes directly. *Drosophila* PGRP-SB1 could kill *Bacillus megaterium* in vitro [31]. Recombinant *B. mori* PGRP-S5 exhibited strong antibacterial activity against gram-positive and gram-negative bacteria [32]. The role of the PGRP family in host defense is relatively well-characterized in *D. melanogaster*. The specific functions of PGRPs in other insects are still largely unknown.

The Asian corn borer, *Ostrinia furnacalis* (Guenée), is an important insect pest in Asia, causing serious damage to corn, sorghum, millet, and other crops [33]. So far, the understanding of pattern recognition receptors, including PGRPs, in this pest is nearly nonexistent. In previous work, we obtained a transcriptome dataset from *O. furnacalis* larvae and identified 10 putative PGRP transcripts [34]. Here, we selected one of these 10 transcripts, designated as PGRP1, for further investigation. Our results indicate that the transcript levels of *PGRP1* were increased upon bacterial and fungal challenges. Recombinant *O. furnacalis* PGRP1 could bind to *E. coli*, *M. luteus*, and *B. bassiana* and further induced their agglutination. Moreover, *O. furnacalis* PGRP1 bound to the Lys-type peptidoglycan from *M. luteus* with a higher affinity than to the DAP-type peptidoglycan from *E. coli*. The injection of recombinant PGRP1 into larvae led to a significant increase in the synthesis of some antimicrobial peptides (AMPs), including *attacin*, *cecropin-4*, *gloverin-1*, and *moricin-4*.

## 2. Results

### 2.1. cDNA Cloning and Sequence Analysis of O. furnacalis PGRP1

We identified 10 putative PGRP transcripts from the *O. furnacalis* transcriptome dataset [34]. Among these, the one designated as OfPGRP1 was selected for further investigation. Since the identified *OfPGRP1* sequence was predicted to be full-length, we designed specific primers containing the whole open reading frame (ORF) and conducted PCR to amplify the full-length cDNA sequence of PGRP1 with cDNA from *O. furnacalis* larvae as a template. The ORF of *O. furnacalis PGRP1* encodes a 194-amino acid residues, including a predicted 20-residue secretion signal peptide. The calculated molecular mass and isoelectric point of the mature protein were 20.6 kDa and 7.93, respectively.

**Figure 1:**
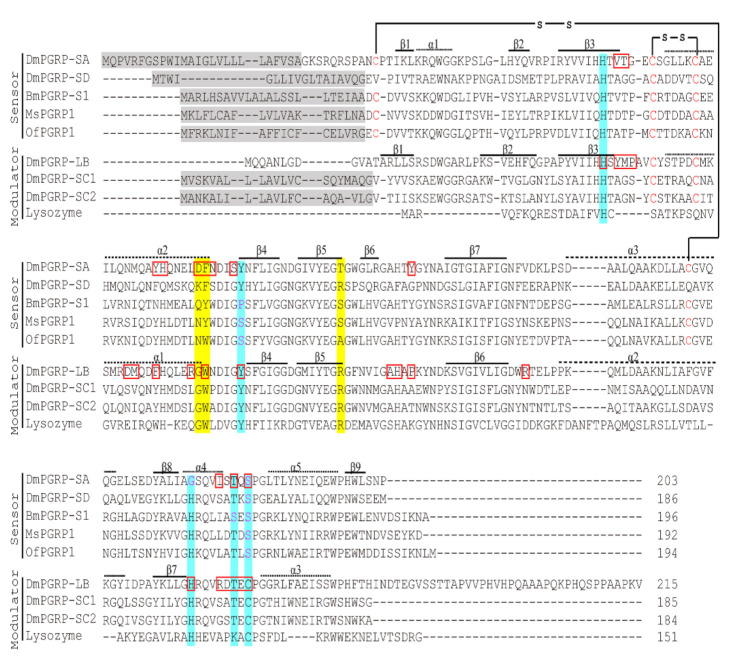
Sequence alignment of *O. furnacalis* PGRP1 with PGRPs from other insects. The GenBank accession numbers for the sequences used are as follows: DmPGRP-SA, Q9VYX7; PGRP-SD, Q9VS97; PGRP-SC1, Q9V3B7; PGRP-SC2, Q9V4X2; PGRP-LB, Q9VGN3; BmPGRP-S1, NM_001043371; MsPGRP1, AF413068; Lysozyme, NP_041973. The predicted signal peptides are shaded in gray. The conserved Cys residues for disulfide bonds are indicated in red. Residues involved in the binding pocket in DmPGRP-SA and -LB are labeled in red boxes. Residues essential for T7 lysozyme activity are shaded in blue. The putative peptidoglycan-recognition positions are shaded in yellow. Secondary structures are assigned the above sequences from the N to C termini of amino acid sequences.

A database search indicated that among those PGRPs with known functions, *O. furnacalis* PGRP1 is the most similar in its amino acid sequence to *B. mori* PGRP-S1 and *M. sexta* PGRP1, with 57% identity with each. The deduced amino acid sequences of *O. furnacalis* PGRP1 were aligned with well-characterized PGRPs from *D. melanogaster*, *B. mori*, and *M. sexta*, and T7 lysozyme. The alignments revealed that two of the five key residues responsible for zinc-binding and amidase activity (His18, Tyr47, His123, Lys129 (Thr in *D. melanogaster* PGRP-LB), and Cys131) in T7 lysozyme were substituted, from Tyr47 to Ser86 and from Cys131 to Ser168 in *O. furnacalis* PGRP1 (Figure 1). This suggests that *O. furnacalis* PGRP1 might lack catalytic amidase activity and fail to hydrolyze peptidoglycan, and it may therefore potentially act as a sensor to recognize the invading microorganisms and initiate the immune response in *O. furnacalis*. Furthermore, two disulfide bonds that contribute to the structural integrity (Cys74–Cys80 and Cys37–Cys160 in *D. melanogaster* PGRP-SA, Cys58–Cys64 and Cys22–Cys144 in *M. sexta* PGRP1) are completely conserved in *O. furnacalis* PGRP1 (Cys58–Cys64 and Cys22–Cys144) (Figure 1).

### 2.2. Expression Profiles of O. furnacalis PGRP1

**Figure 2:**
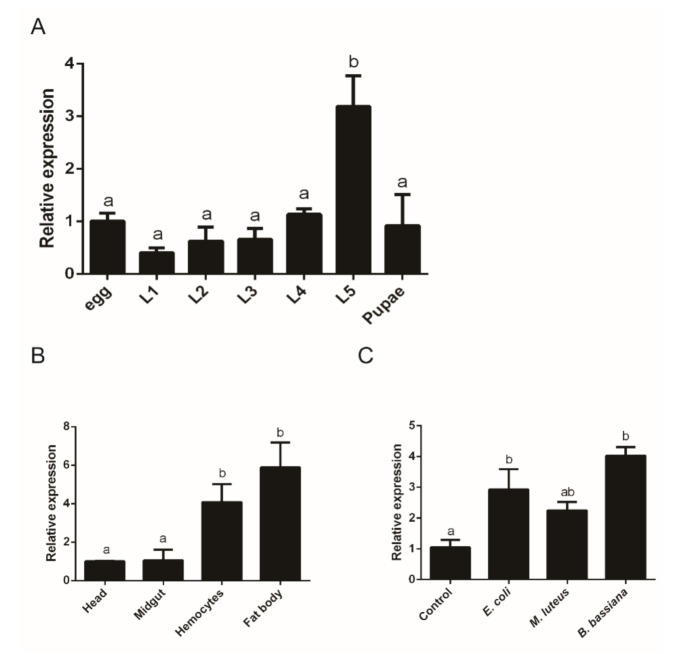
Expression profile analysis of *O. furnacalis PGRP1*. (**A**) Expression profiles of *O. furnacalis PGRP1* at different stages of development. RNA was extracted from the whole bodies of collected eggs; first-instar (L1), second-instar (L2), third-instar (L3), fourth-instar (L4), and fifth-instar (L5) larvae; and pupae. The *rpL8* gene was used as an internal control. (**B**) Expression profiles of *O. furnacalis PGRP1* in various tissues. Hd, head; Mg, midgut; Hc, hemocytes; Fb, fat body (*O. furnacalis*). (**C**) Expression profiles of *O. furnacalis PGRP1* upon microbial challenge. On day 0, fifth-instar larvae were infected with PBS (Control), *E. coli*, *M. luteus*, or *B. bassiana*. RNA was prepared from the whole bodies 24 h after injection. The *rpL8* gene was used as an internal standard to indicate a consistent total mRNA amount. Bars labeled with different letters are significantly different (one–way ANOVA, followed by Tukey’s multiple comparisons test, *p* < 0.05).

We analyzed the mRNA levels of *O. furnacalis PGRP1* in various development stages, different tissues, and different pathogen challenges using quantitative RT-PCR methods. As shown in Figure 2A, the *O. furnacalis PGRP1* transcript was significantly higher in the fifth-instar larvae than in insects at other developmental stages. No obvious difference was observed in the transcripts from other developmental stages except the fifth-instar larvae (Figure 2A). Among the different tissues, the fat bodies and hemocytes had higher transcript levels of *PGRP1* than did the head and midgut (Figure 2B).

To determine the expression patterns of *O. furnacalis PGRP1* after exposure to microbial elicitors, we analyzed its transcript level after *O. furnacalis* larvae were injected with *E. coli*, *M. luteus*, *B. bassiana*, or PBS as a control. The results from the qPCR assay indicated that the mRNA abundance of *O. furnacalis PGRP1* increased significantly in the larvae challenged by *E. coli* or *B. bassiana* conidia (Figure 2C).

### 2.3. Production of Recombinant PGRP1 (rPGRP1)

**Figure 3:**
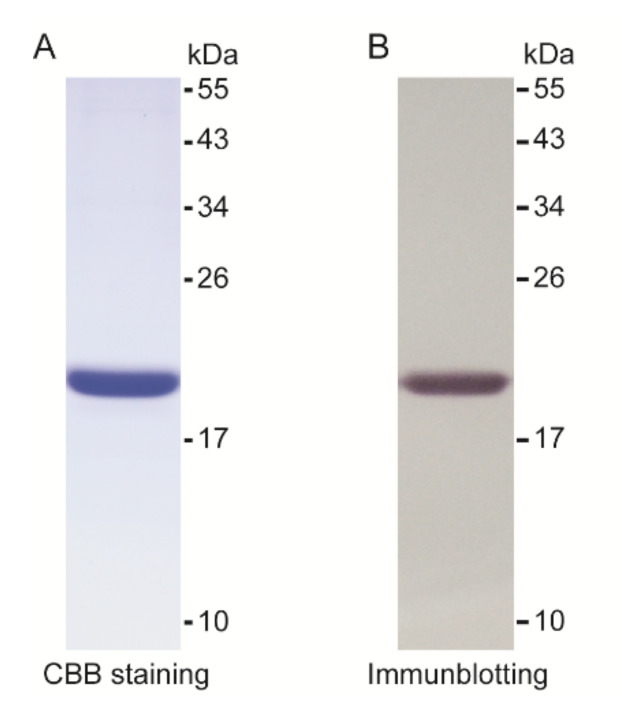
SDS-PAGE (**A**) and immunoblot (**B**) analysis of purified PGRP1. The purified recombinant PGRP1 was separated by 15% SDS-PAGE followed by Coomassie Brilliant Blue staining (lane 1, 3 μg) or immunoblotting (lane 2, 0.6 μg) with mouse monoclonal anti-polyhistidines as primary antibodies. The sizes and positions of the molecular weight standards are indicated on the right.

In order to investigate the function of *O. furnacalis* PGRP1, we expressed the recombinant PGRP1 in *E. coli* with an amino-terminal hexahistidine tag. The soluble rPGRP1 was purified by nickel-affinity chromatography followed by Q-Sepharose anion exchange chromatography. It has an apparent molecular mass of approximately 21 kDa (Figure 3A and Figure S1). This was approximately equal to the calculated molecular mass (20.6 kDa). The purified recombinant PGRP1 could be strongly recognized by commercial mouse monoclonal antibodies against hexahistidine tags (Figure 3B).

### 2.4. O. furnacalis PGRP1 Bound to Bacteria and Fungi and Caused Agglutination

**Figure 4:**
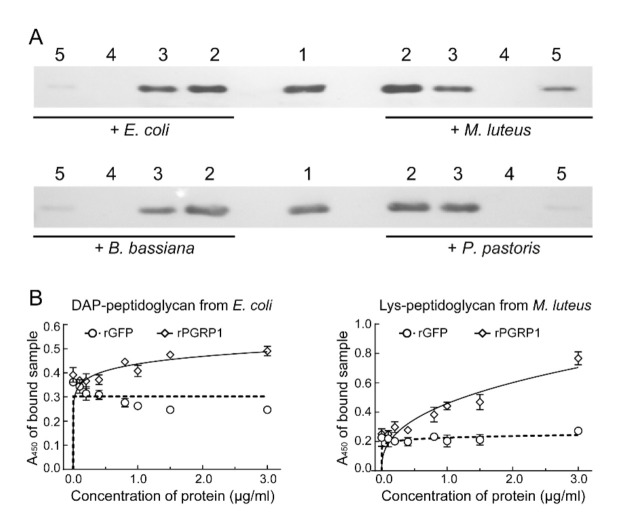
Binding of recombinant PGRP1 to different microorganisms (**A**) and peptidoglycans (**B**). (**A**) Recombinant PGRP1 was incubated with *E. coli, M. luteus, B. bassiana*, and *P. pastoris*. After incubation for 10 min, the incubated mixtures were pelleted and washed three times, and the pellet was resuspended. The samples from each step were separated by 15% SDS-PAGE and visualized by immunoblotting with mouse monoclonal anti-polyhistidine as the primary antibody. Lane 1, recombinant PGRP1 (0.1 μg); lane 2, total mixture before centrifugation; lane 3, the supernatant after centrifugation (unbound fraction); lane 4, the fraction after washing three times; lane 5, the suspended pellet (bound fraction). (**B**) Binding of PGRP1 to peptidoglycans. Microtiter plates were coated by DAP-type peptidoglycan from *E. coli* K12 or Lys-type peptidoglycan from *M. luteus*. Increasing concentrations of rPGRP1 were added to the coated wells (the same amount of rGFP was added as a control). The binding assay was performed as described in “Materials and methods”. Each point represents the mean ± S.D. (*n* = 3). The solid lines represent the nonlinear regression calculation of the one-site binding curve.

We examined whether or not the recombinant PGRP1 could bind to bacteria or fungi. As shown in Figure 4A, PGRP1 exhibited, to some extent, affinity to the bacteria *E. coli* and *M. luteus*, the entomopathogenic fungus *B. bassiana*, and the yeast *P. pastoris*. Under the same experimental conditions, the intensity of the band corresponding to the PGRP1 bound to *M. luteus* was the highest, suggesting that more recombinant PGRP1 bound to *M. luteus* than to the other three microbes (Figure 4A). Additionally, we performed an enzyme-linked immunosorbent assay to measure the binding capacity of recombinant PGRP1 to different types of peptidoglycans. Recombinant PGRP1 or GFP (as a control) at different concentrations was added to the wells of a microtiter plate coated with DAP-type peptidoglycan from *E. coli* or Lys-type peptidoglycan from *M. luteus*, respectively. After an incubation period and washing, the bound PGRP1 or GFP was detected using antiserum against the polyhistidine tag. As increasing amounts of PGRP1 were added, more PGRP1 bound to immobilized peptidoglycan (Figure 4B). Moreover, nonlinear regression analysis of the binding data indicated that recombinant PGRP1 bound to Lys-type peptidoglycan from *M. luteus* (*K*_d_ = 66.2 μg/mL) with a higher affinity than to DAP-type peptidoglycan from *E. coli* (*K*_d_ = 150.6 μg/mL) (Figure 4B).

**Figure 5:**
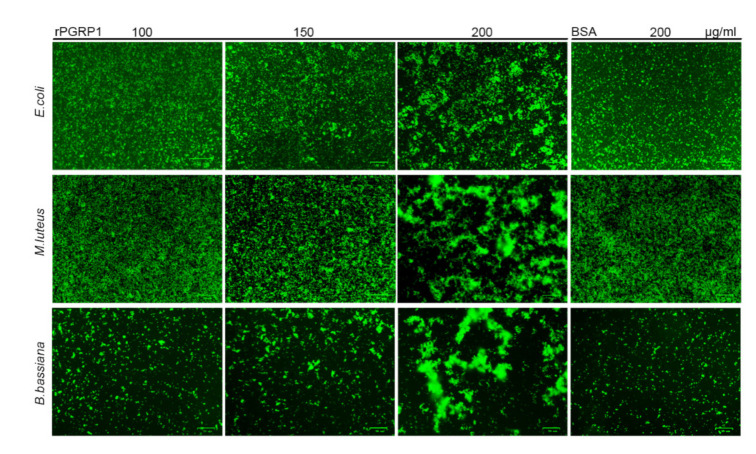
Agglutination of microorganisms by recombinant PGRP1. Different concentrations of recombinant PGRP1 (100 μg/mL, 150 μg/mL, and 200 μg/mL) were incubated with acridine orange-stained *E. coli* (2 × 10^8^ cells/mL), *M. luteus* (2 × 10^8^ cells/mL), or *B. bassiana* (1 × 10^5^ conidia/mL), respectively, in PBS for 45 min at room temperature. BSA (200 μg/mL) instead of rPGRP1 was used as a control. The agglutination of microbial cells was then observed under invert fluorescence microscopy.

In addition, we tested whether the binding of recombinant PGRP1 to microorganisms could result in the agglutination of the corresponding microbes. The results showed that the recombinant PGRP1 caused increased agglutination of *E. coli*, *M. luteus*, and *B. bassiana* compared with BSA (Figure 5). Moreover, the agglutination occurred in a concentration-dependent manner. The agglutination was negligible with recombinant PGRP1 at a concentration of 100 μg/mL, but PGRP1 at the final concentration of 200 μg/mL led to pronounced agglutination of the tested bacteria or fungi (Figure 5).

### 2.5. Recombinant PGRP1 Increased PO Activity of O. furnacalis Plasma

**Figure 6:**
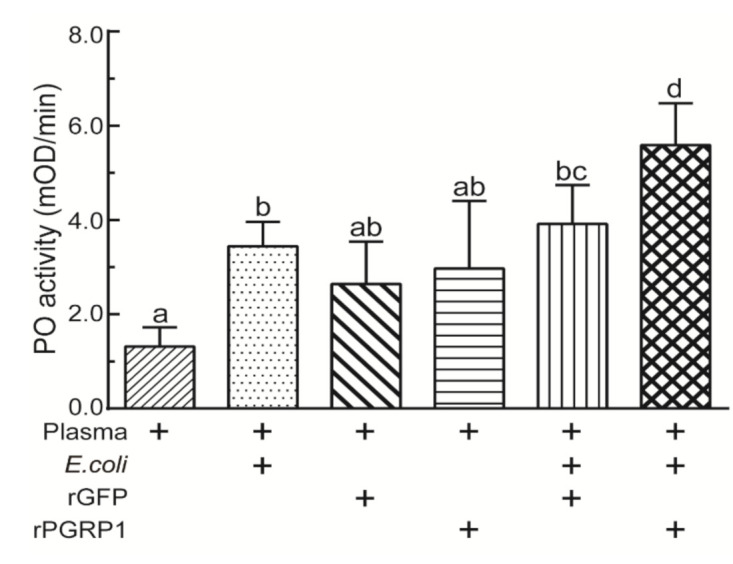
Stimulation of PPO activation in *O. furnacalis* plasma upon the addition of recombinant PGRP1. Recombinant PGRP1 (100 μg/mL) or rGFP (as a control) were mixed with plasma from *O. furnacali* fifth-instar day 0 larvae and incubated in the presence or absence of *E. coli* (1.6 × 10^8^ cells/mL) at room temperature for 10 min. Details of the reaction mixtures are provided in the figures. The bars represent mean ± S.D. (*n* = 3). Bars labeled with different letters are significantly different (one–way ANOVA, followed by Tukey’s multiple comparisons test, *p* < 0.05).

We also investigated whether the binding of bacteria by PGRP1 may be involved in the PPO activation system in *O. furnacalis*. Purified rPGRP1 or rGFP (as a control), alone or in combination with *E. coli*, was incubated with *O. furnacalis* plasma, and PO activity was measured. The plasma from fifth-instar larvae had basal PO activity (Figure 6). The incubation of the *E. coli* elicitor with plasma led to a significant increase in PO activity. The addition of rPGRP or rGFP alone to plasma failed to activate the PPO pathway. However, supplementing the plasma with rPGRP1 combined with *E. coli* resulted in a significant increase compared with that caused by *E. coli* alone or the mixture of rGFP and *E. coli* (Figure 6). This suggests that rPGRP1 is involved in PPO activation, possibly through the recognition of invaders such as *E. coli*.

### 2.6. O. furnacalis PGRP1 was Related to the Expression of AMP Genes

**Figure 7:**
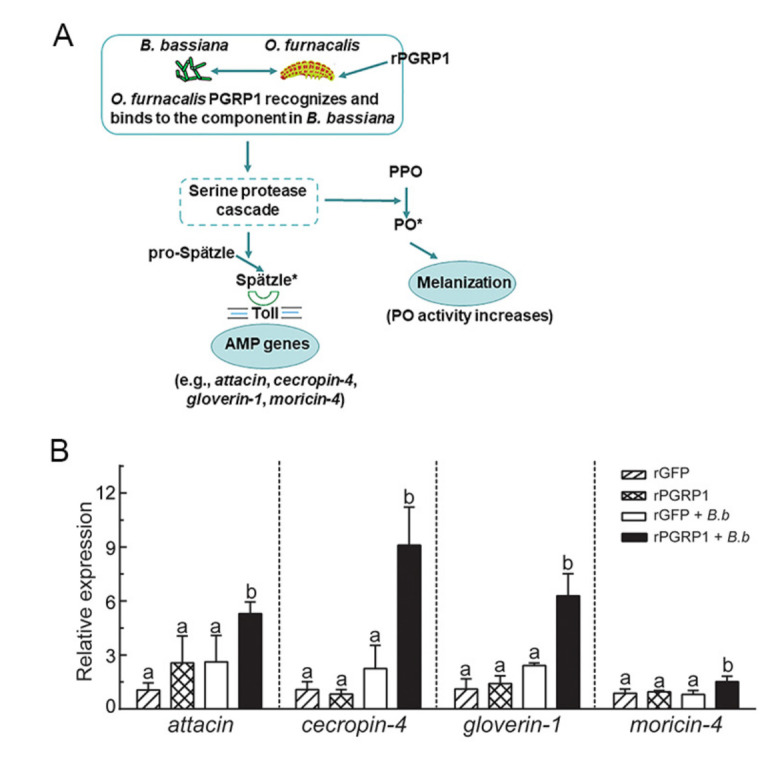
(**A**) Schematic diagram to explain how the injection of PGRP1 and *B. bassiana* boosted the expression of AMPs. The recognition and binding of *O. furnacalis* PGRP1 to PAMP in *B. bassiana* stimulated the activation of serine protease cascades. In one pathway, it resulted in the conversion of PPO into PO, whose activity increased greatly. In the other pathway, it led to the activation of pro-Spätzle, which further bound to the Toll receptor to initiate the expression of AMP genes, including *attacin*, *cecropin-4*, *gloverin-1*, *moricin-4*, etc. (**B**) Induced expression of AMP genes in *O. furnacalis* upon the injection of recombinant PGRP1. Purified recombinant PGRP1 or GFP (2 μg) were injected into the day 0 fifth-instar larvae of *O. furnacalis.* For some treatments, the larvae were injected again 30 min later with *B. bassiana* conidia suspension (5 × 10^3^ conidia) or PBS as a control. Total RNA samples were isolated from the whole body at 24h after injection for qRT-PCR analysis. *O. furnacalis rpL 8* was used as an internal standard to normalize the templates. The bars represent the mean ± S.D. (*n* = 3). Bars labeled with different letters are significantly different (analysis using one-way ANOVA followed by Newman–Keuls test, *p* < 0.05).

To examine whether *O. furnacalis PGRP1* is also involved in another immune response, the induction of AMP production, we increased the concentration of PGRP1 in *O. furnacalis* hemolymph via the injection of purified rPGRP1 or rGFP into larvae and then determined the transcript levels of some AMP genes with quantitative RT (qRT) -PCR methods. As shown in Figure 7, when *O. furnacalis* larvae were injected with recombinant GFP and PGRP1 proteins, no significant difference was observed in the transcript levels of AMP genes, including *attacin*, *cecropin-4*, *gloverin-1*, and *moricin-4*. However, when larvae injected with rPGRP1 were injected 30 min later with *B. bassiana* conidia, the mRNA levels of all four tested AMP genes increased significantly compared with the control larvae injected with rGFP and *B. bassiana.* The increase in the *cecropin-4* transcripts was especially significant (*p* < 0.05). The results suggest that PGRP1 is involved in a pathway leading to *B. bassiana*-induced expression of AMP genes in *O. furnacalis*.

## 3. Discussion

PGRPs are a category of important PRRs and play various physiological roles in animals, including humans and insects. In this study, we selected a PGRP with a high binding potential (PGRP1) from the *O. furnacalis* transcriptome for functional characterization. The transcript levels of PGRP1 were increased after a microbial challenge. Recombinant PGRP1 could bind to *E. coli*, *M. luteus*, and *B. bassiana* and further induced their agglutination. This binding might be related to PPO activation and AMP synthesis in the *O. furnacalis* immune response.

It seems that PGRPs are widely distributed among animals, including insects, echinoderms, mollusks, and vertebrates, but not in lower metazoa (nematodes) or plants [35]. PGRP genes have been identified in all sequenced insects, including *Aedes agypti*, *A. gambiae*, *Apis mellifera*, *B. mori*, *D. melanogaster*, *M. sexta*, and *T. castaneum* [17] (and references within). In the Asian corn borer *O. furnacalis*, we identified 10 putative PGRP sequences from the transcriptome [34]. Here, we cloned the full-length cDNA for one of these 10 transcripts, *PGRP1*. The open reading frame of *O. furnacalis PGRP1* encodes a 194-amino acid residue protein with a 20-residue signal peptide. Therefore, *O. furnacalis* PGRP1 is an extracellular protein and performs functions extracellularly. Sequence analysis indicated the PGRP1 contains a typical PGRP domain, which possesses five key amino acid residues (His43, Tyr78, His152, Thr158, and Cys160 in PGRP-LB) essential for amidase activity and several relatively conserved amino acid residues for the binding groove [36]. Multiple sequence alignment indicated that Tyr78 and Cys160 (PGRP-LB numbering) were replaced by Ser86 and Ser168 in PGRP1, respectively (Figure 1). We speculated that *O. furnacalis* PGPR1 lacks amidase activity because of the replacement of amino acid residues at the above essential positions. Phylogenetic analysis (Figure S2) also revealed that PGRP1 clustered together with *B. mori* PGRP-S1 and *M. sexta* PGRP1, which both lack amidase activity, and participated in PPO activation by acting as a recognition receptor [13,28]. The antibacterial assay indicated that *O. furnacalis* PGRP1 had no direct antibacterial activity (Figure S3), possibly because of the deficiency of the amidase activity. We anticipated that *O. furnacalis* PGPR1 may act as a non-catalytic receptor-type PGRP like its orthologs *B. mori* PGRP-S1 and *M. sexta* PGRP1 but not as an amidase-type PGRP.

The expression profile of *O. furnacalis PGPR1* was also consistent with that of *M. sexta PGRP1*. The latter was mainly expressed in the fat bodies of naïve larvae [13]. In our study, we found that the *O. furnacalis PGPR1* mRNA level was significantly higher in the fat bodies (the equivalent of the liver in mammals) than in the hemocytes (Figure 2B). Similarly, *BmPGRP-S1* in the fat bodies exhibited the highest expression level among the immune organs of the silkworm, to which *O. furnacalis PGPR1* is most similar in deduced amino acid sequences (Figure S2). Moreover, *PGRP1* transcription significantly increased upon immune stimulation from bacteria or fungi (Figure 2C). This immune-responsive expression profile has been observed in *B. mori PGRP-S5* [32], *M. sexta* PGRP1 [28], and *Helicoverpa armigera PGRP-B* and *PGRP-C* [37]. It is likely that the mRNA of *O. furnacalis* PGPR1 increased greatly upon immune challenge so that there would be enough PGRP1 to exert its physiological function after *O. furnacalis* was infected.

Recombinant *O. furnacalis* PGPR1 exhibited some potential for binding to the bacteria *M. luteus* and *E. coli*, the fungus *B. bassiana*, and the yeast *P. pastoris*, especially *M. luteus* (Figure 4A). It was not surprising that PGRP1 had a board-spectrum affinity for both bacteria and fungi. *M. sexta* PGRP1 also bound to multiple microbes, including *M. luteus*, *Staphylococcus aureus, Bacillus megaterium*, and *Bacillus subtilis* [28]. *Crassostrea gigas* PGRP-S2 could bind to *E. coli*, *Vibrio* *anguillarum*, *S. aureus*, and *Yarrowia* *lipolytica* [38]. *Branchiostoma japonicum* PGRP-S could bind to *S. aureus*, *E. coli*, and *P. pastoris* [39]. On the other hand, the binding of PGRP to bacteria starts with its recognition of peptidoglycans, which are cell components of bacteria. Peptidoglycans are diversified depending on the bacterial species; many gram-negative bacteria and some gram-positive bacteria, such as the *Bacillus* species, have DAP-type peptidoglycans, while many gram-positive bacteria have Lys-type peptidoglycans [23]. PGRPs discriminate between peptidoglycans from different bacteria species through the distinct features of their stem peptides, such as the amino acid composition [40]. Insect PGRPs contain approximately sixteen residues that contact peptidoglycans. Structural analysis, along with site-directed mutagenesis, indicated that several residues in the binding groove (shaded in yellow in Figure 1) may participate in the differential recognition of DAP- and Lys-type peptidoglycans [41]. For instance, *D. melanogaster* PGRP-SA and -SD contain Asp70 (Lys61 in PGRP-SD)–Phe71 (Phe62 in PGRP-SD) and recognize Lys-type peptidoglycans. In comparison, *D. melanogaster* PGRP-LB, -LCx, and -LE have Gly and Trp at the corresponding positions and recognize DAP-type peptidoglycans [42]. *M. sexta* PGRP1 has Asn80–Tyr81 at these sites and binds to DAP-type peptidoglycans preferentially [42]. In *O. furnacalis* PGRP1, Asn80–Trp81 is located at these two positions (Figure 1). However, the enzyme-linked immunosorbent assay indicated that *O. furnacalis* PGRP1 had a higher affinity to Lys-type peptidoglycans (*K*_d_ = 66.2 μg/mL) than to DAP-type peptidoglycans (*K*_d_ = 150.6 μg/mL μg/mL) (Figure 4B). A possible explanation is that sequence variations at these positions in PGRPs affect the binding ability to different peptidoglycans [43]. Additionally, the Arg254 in the *D. melanogaster* PGRP-LE is believed to be responsible for DAP-type peptidoglycan recognition [43]. This Arg is highly conserved in *Drosophila* and human PGRPs, which recognize DAP-PGRPs, but is not always present in PGRPs that recognize Lys-type peptidoglycans (Figure 1). However, *D. melanogaster* PGRP-SD possesses an Arg at this site but binds to Lys-type peptidoglycans more preferentially than to DAP-type peptidoglycans [44]. *M. sexta* PGRP1 has Ser100 at the position of Arg but exhibited a higher binding tendency to DAP-type peptidoglycans [42]. In *O. furnacalis* PGRP1, this Arg is replaced by Ala100, different from any aligned PGRPs with known binding affinities (Figure 1). It is difficult to make predictions for the binding preference of *O. furnacalis* PGRP1 based only on its amino acid sequence. Further experiments are underway to determine its binding specificities.

As a family of pattern recognition proteins, PGRPs function as sensors to detect DAP- or Lys-type peptidoglycans from different bacteria during the early stage of infection, and the recognition activates a series of immune pathways, including the Toll, Imd, and PPO activation pathways [45]. In addition to the examples listed above, including *D. melanogaster* PGRP-SA, -SD, and -LE [46]; *B. mori* PGRP-S1 [13]; *M. sexta* PGRP1 [28]; and *T. molitor* PGRP-SA [29], the recognition of peptidoglycans by PGRP-SA in *Antheraea pernyi* resulted in PPO activation as well as AMP production [28,47]. Our data indicated that the addition of *O. furnacalis* PGRP1 to the larval plasma enhanced PPO activation in the presence of a microbial elicitor (Figure 6). Furthermore, injection of recombinant PGRP1 into *O. furnacalis* larvae resulted in a significant increase of the transcript levels of several AMP genes when the larvae were challenged by *B. bassiana* (Figure 7). However, unlike *D. melanogaster* PGRP-SB1 [31] and *H. armigera* PGRP-B and -C [37], *O. furnacalis* PGRP1 had no direct antibacterial activity (Figure S3). Taken together, the results suggest that in the *O. furnacalis* immune response, PGRP1 only serves as a receptor for peptidoglycan recognition, which initiates the PPO activation pathway and AMP synthesis, and it may not act as an effector to directly kill the invading pathogens. The detailed function mechanism of PGRP1 remains unknown and needs further investigation.

## 4. Materials and Methods

### 4.1. Biological Materials

Asian corn borers, *O. furnacalis* (Guenée), were reared on an artificial diet at 28 °C under a relative humidity of 70–90% and a photoperiod of 16 h light and 8 h darkness. *Beauveria bassiana* (strain 252) was cultured on potato dextrose agar (PDA) plates at 25 °C and a relative humidity of 80%. For *Escherichia coli* (strain DH5α) and *Micrococcus luteus* (strain CMCC(B)28001), a single colony was grown in Luria–Bertani (LB) plates overnight at 37 °C and subcultured in the same medium until OD_600_ was close to 0.8. *Pichia pastoris* (strain AH109) was grown in a yeast extract peptone dextrose (YEPD) medium at 28 °C until OD_600_ was close to 0.8. After centrifugation at 4000 rpm and washing with phosphate-buffered saline (PBS) three times, the bacteria or fungi were resuspended in PBS for use.

### 4.2. Sequence Analysis and Comparison

The analysis of the deduced amino acid sequences, including the prediction ofmolecular weightand isoelectric point, was performed in the EXPASY (Expert Protein Analysis System) (https://www.expasy.org/resources/compute-pi-mw accessed on 17 May 2021). Signal peptides were predicted by the Signal P 5.0 server (http://www.cbs.dtu.dk/services/SignalP/ accessed on 17 May 2021). Conserved domains and transmembrane regions were predicted in SMART (http://smart.embl-heidelberg.de/smart/set_mode.cgi accessed on 17 May 2021). Multiple sequence alignment of PGRP1 with other insect PGRPs was performed in DNAMAN.

### 4.3. Expression Profile Analysis of O. furnacalis PGRP1

To investigate the changes of *O. furnacalis PGRP1* transcript levels in several developmental stages, total RNA samples were individually prepared (*n* = 3) from three different stages, including the egg, first- to fifth-instar day 0 larvae, and pupa, using TRIzol Reagent (TIANGEN, Biotech Co. Ltd., Beijing, China). One microgram of RNA samples from 3 individual RNA samples in each stage was treated equally with DNase I (TIANGEN, Biotech Co. Ltd., Beijing, China) and converted into first-strand cDNA with an oligo (dT) primer following the instructions for QuantScriptRT Kit (TIANGEN, Biotech Co. Ltd., Beijing, China). The cDNA products from 3 biological replicates were independently diluted 10-fold for use as template in the qRT-PCR experiments. Specific primers were designed and are listed in Table S1. *O. furnacalis* ribosomal protein L8 (*rpL8*) was used as an internal standard to adjust the template amounts in preliminary PCR experiments. The thermal cycle conditions for qRT-PCR were 95 °C for 30 s, followed by 40 cycles of 95 °C for 5 s, 60 °C for 34 s, and 68 °C for 30 s, and ending with a melting curve generation (60 °C to 95 °C in increments of 0.5 °C every 5 s). The relative expression levels of genes were calculated using the 2^−^^ΔΔCt^ method. Each qRT-PCR assay was performed with 3 biological and 2 technical replicates.

To determine the expression patterns of *O. furnacalis PGRP1* in different tissues, total RNA samples were isolated separately from combined heads, midguts, fat bodies, and hemocytes of fifth-instar larvae. The synthesis of first-strand cDNA and RT-PCR analysis was performed as described above. To check the expression profiles of *O. furnacalis PGRP1* after different microbial injections, fifth-instar day 0 larvae were injected into the hemocoel with 2 μL of sterile PBS containing formaldehyde-killed *E. coli* (5 × 10^3^ cells/μL) and dried *M. luteus* (5 μg/μL) or *B. bassiana* conidial suspension (3 × 10^4^ conidia/μL) or sterile PBS as a control. After 24 h, three larvae each from the challenged or control group were collected, and total RNA samples were prepared from each larva. Subsequently, the qRT-PCR analysis was conducted as described above.

### 4.4. Production and Purification of Recombinant PGRP1 (rPGRP1)

A pair of specific primers, listed in Table S1, was designed to amplify the sequence encoding the mature peptide of PGRP1. The forward primer included an *Nco* I site, which provided the start codon, followed by one codon for the glycine residue and six codons for the histidine residues. The reverse primer contained a *Not* I site at the 3′-end of the stop codon. The PCR products were ligated into the pMD19-T vector and then digested with *Nco* I and *Not* I. Then, the digested cDNA fragment was subcloned into the same restriction sites of the expression vector pET28a (Novagen). In addition, the *E. coli* strain BL21 (DE3) was transformed with the resulting PGRP1/pET28a or GFP/pET28a plasmid gifted by Zhejiang University. For recombinant protein expression, a single clone was incubated at 37 °C in an LB medium containing 50 μg/mL kanamycin. When the OD_600_ reached 0.8, isopropyl β-D-thiogalactoside (IPTG) was added at a final concentration of 0.1 mM, and recombinant protein was expressed for 12 h at 20 °C and 200 rpm. Bacterial cells were harvested by centrifugation at 5000 × *g* for 20 min and resuspended with lysis buffer (50 mM sodium phosphate, 300 mM NaCl, 10 mM imidazole; pH 8.0). Cells were lysed by sonication, and the supernatant was obtained by centrifugation. Then, the soluble PGRP1 or GFP was purified as described previously [48].

### 4.5. Binding of rPGRP1 to Different Microorganisms

*E. coli, M. luteus, B. bassiana*, and yeast (*P. pastoris*) were used to test the binding activity of rPGRP1. Bacteria and yeast in the mid-logarithmic phase were harvested by centrifugation at 5000 rpm for 5 min. After washing with PBS three times, the cells were resuspended in PBS. A *B. bassiana* conidia suspension (1 × 10^5^ conidia/mL) was prepared as described previously [34]. Then, 40 μL of the microbial suspension was separately incubated with 40 μL of purified rPGRP1 (200 μg/mL) at room temperature with gentle rotation. Ten minutes later, the mixture was centrifuged at 8000 × *g* for 10 min, and the supernatant was saved as the unbound sample. The resulting cell pellet was washed three times with 200 μL PBS, and the supernatant from each wash step was taken as the wash sample. The final pellet was resuspended with 20 μL of 2× SDS sample buffer. The collected samples from each step were separated by 15% SDS-PAGE and subjected to immunoblotting analysis using 1:2000 diluted mouse monoclonal anti-polyhistidine antibody and horse-anti-mouse IgG conjugated to alkaline phosphatase [49].

### 4.6. Binding of rPGRP1 to Peptidoglycans

The binding of rPGRP1 to different types of peptidoglycans was examined using the enzyme-linked immunosorbent assay (ELISA). The Lys-type peptidoglycan from *M. luteus* (53243, Sigma) and the DAP-type peptidoglycan from *E. coli* K12 (tlrl-pgnek, InvivoGen) were diluted to 40 μg/mL with water. ELISA plates (F605032, Sangon Biotech) were coated with each peptidoglycan (2 μg/well) overnight. Then, all wells were washed four times with TBS containing 0.1% Tween-20 and blocked with TBS containing 10 mg/mL BSA at 37 °C for 2 h. The plate was washed three times again with TBS containing 0.1% Tween-20. Then, rPGRP1 at different concentrations was added to each well and incubated at 37 °C for 2 h (recombinant GFP proteins were used as controls). After washing three times, the plate was incubated with mouse monoclonal anti-polyhistidine antibody (1:2000 in TBS containing 1 mg/mL BSA, 100 μL/well) at 37 °C for 2 h. The plate was washed three times and incubated with goat anti-rabbit IgG conjugated to HRP (1:5000 in TBS containing 1mg/mL BSA, 100 μL/well) at 37 °C for 2 h. After washing three times again, the EL-TMB Chromogenic Reagent kit (C520026, Sangon Biotech) was used for color development. Absorbance at 450 nm was measured on a microplate spectrophotometer (PowerWave HT, Biotek).

### 4.7. Microbial Agglutination Assay

*E. coli, M. luteus*, and *B. bassiana* were used for the agglutination assay as described previously [40]. *E. coli* and *M. luteus* were harvested in the mid-logarithmic phase by centrifugation at 5000× *g* for 5 min. After staining with acridine orange, the cells were washed three times with PBS and resuspended in PBS at a concentration of 2 × 10^8^ cells/mL. A *B. bassiana* conidia suspension (1 × 10^5^ conidia/mL) was prepared as described previously. The diluted microbial suspension (30 μL) was separately mixed with different concentrations of rPGRP1 (final concentrations of 100 μg/mL, 150 μg/mL, and 200 μg/mL), and Bovine serum albumin (BSA, 200 μg/mL) instead of rPGRP1 was used as a control. After incubation in a 96-well plate at room temperature for 45 min, agglutination reactions were observed under an inverted fluorescence microscope (Bio-rad, USA).

### 4.8. Stimulation of PPO Activation by rPGRP1 in O. furnacalis Larval Plasma

One microliter of recombinant PGRP1 (100 μg/mL) or GFP (used as a control) was mixed with 1 μL of plasma from *O. furnacali* fifth-instar day 0 larvae. Then, the mixtures were incubated in the presence or absence of *E. coli* (1.6 × 10^8^ cells/mL) at room temperature for 10 min. Details of the reaction mixtures are provided in the figures and corresponding legends. Each treatment underwent three replications. One unit of PO activity was defined as the amount of enzyme producing an increase in absorbance (OD_490_) of 0.001 per min.

### 4.9. Analysis of the Expression of Antimicrobial Peptide Genes after Injection of rPGRP1

Purified recombinant PGRP1 or GFP (2 μg) were injected into the day 0 fifth-instar larvae of *O. furnacalis.* In some experiments, the larvae were given a second injection 30 min later of *B. bassiana* conidia suspension (5 × 10^3^ conidia) or sterile PBS as control. After 24 h, total RNA samples were prepared from each larva as described above. DNase I-treated RNA (1 μg) was converted into first-strand cDNA using FastQuant RT Kit (TIANGEN Biotech, China). The cDNA products were diluted 10-fold for use as template. The quantitative real-time PCR (qRT-PCR) was performed with TB Green^®^ Premix EX Taq^TM^ (TaKaRa Biotechnology Co. Ltd., Dalian, China) on the Applied Biosystems^®^ 7500 Real-Time PCR System (Life Technologies, Foster City, CA, USA), according to the manufacturer’s instructions. *O. furnacalis* ribosomal protein L8 (*rpL8*) was used as an internal standard to normalize the expression level. The thermal cycle conditions for qRT-PCR were 95 °C for 30 s, followed by 40 cycles of 95 °C for 5 s, 60 °C for 34 s, and 68 °C for 30 s, and ending with a melting curve generation (60 °C to 95 °C in increments of 0.5 °C every 5 s). The relative expression levels of genes were calculated using the 2^−ΔΔCt^ method. Each qRT-PCR experiment was performed with 3 biological and 2 technical replicates.

## Supplementary Materials

The following are available online at https://www.mdpi.com/article/10.3390/ijms22158198/s1, Figure S1: SDS-PAGE analysis of partly purified PGRP1 after Ni-affinity chromatography (A, B) and ion-exchange chromatography with Q-Sepharose Fast Flow (C). The purified recombinant PGRP1 was separated by 15% SDS-PAGE followed by Coomassie Brilliant Blue staining. In (A), lanes 1–3: samples washed with buffer (50 mM sodium phosphate, 300 mM NaCl, 30 mM imidazole; pH 8.0); lanes 4–6: samples washed with buffer (50 mM sodium phosphate, 300 mM NaCl, 50 mM imidazole; pH 8.0). In (B), lanes 1–4: samples eluted with buffer (50 mM sodium phosphate, 300 mM NaCl, 100 mM imidazole; pH 8.0); lanes 5–8: samples eluted with buffer (50 mM sodium phosphate, 300 mM NaCl, 250 mM imidazole). In (C), lane 1: flow-through; lane 2: sample washed with buffer (20 mM Tris, 20mM NaCl; pH 8.0); lanes 3–14: samples washed with gradient mixed solutions (20 mM Tris, 20mM NaCl; pH 8.0 and 20 mM Tris, 500 mM NaCl; pH 8.0). The sizes and positions of the molecular weight standards are indicated on the left. Figure S2: Phylogenetic analysis of *O. furnacalis* PGRP1. The amino acid sequences of PGRPsfrom *O. furnacalis* and *D. melanogaster* (Dm, PGRP-SA, CG11709; PGRP-SB1, CG9681; PGRP-SB2, CG9697; PGRP-SC1a, Q9V3B7; PGRPSC1b, Q95SQ9; PGRP-SC2, Q9V4X2; PGRP-SD, CG7496; PGRP-LA, Q9VSV8; PGRP-LB, CG14704; PGRP-LCw, AAL89928; PGRP-LCx, CG4432-PA; PGRP-LE, Q9VXN9), *B. mori* (Bm, PGRP-S1, NP_001036836.1; PGRP-S2, AHJ11178.1; PGRP-S3, NP_001243949.1; PGRP-S4, XP_021205511.1; PGRP-S5, NP_001036858.1; PGRP-S6, XP_012548099.1; PGPR-L1, XP_004929813.1; PGPR-L2, XP_004929814.1; PGPR-L3, XP_028032135.1; PGPR-L4, XP_012549076.1; PGPR-L5, XP_004929948.1; PGPR-L6, XP_004929966.1), *Samia Cynthia ricini* (Sr, PGRP-A, AB232695; PGRP-B, AB232693; PGRP-C, AB232694; PGRP-D, AB291943), *H. armigera* (Ha, PGRP-1, AHK59818.1; PGRP-B, AFP23116; PGRP-C, AFP23117), *M. sexa* (Ms, PGRP-1, AF413068; PGRP-2, ACX49764), *A. gambiae* (Ag, PGRP-LB, XP_003435776), *T. castaneum* (Tc, PGRP-2, XP_969883), *Glossina morsitans* (Gm, PGRP-LB, ABC25064), and *Trichoplusia ni* (Tn, PGRP, AAC31820) were used to build the unrooted tree. Numbers at the branches indicate bootstrap values as percentages of 1000 repetitions. The clade that groups *O. furnacalis* PGRP1 with other PGRPs known as recognition receptors is shaded in yellow. Figure S3: *O. furnacalis* PGRP1 had no antibacterial activity. *M. luteus* was cultured in LB medium at 37 °C overnight to reach 0.6 at OD_600_. The bacteria in 200 μL were then harvested by centrifugation at 5000 × *g* for 5 min at 4 °C and resuspended in 200 μL of sterile PBS. After dilution to 1:100, the suspended *M. luteus* cells (1 mL) were incubated with the purified recombinant PGRP1 (final concentration of 200 μg/mL in LB medium) with gentle shaking for 2, 4, 6, and 8 h at room temperature. The absorbance of the cultures at each time point was measured at 600 nm. Table S1: Nucleotide sequences of primers used for PCR and plasmid construction.

## References

[1] Kingsolver M.B., Hardy R.W. (2012). Making connections in insect innate immunity. Proc. Natl. Acad. Sci. USA.

[2] Kanost M.R., Jiang H.B. (2015). Clip-domain serine proteases as immune factors in insect hemolymph. Curr. Opin. Insect Sci..

[3] Takahashi D., Garcia B.L., Kanost M.R. (2015). Initiating protease with modular domains interacts with beta-glucan recognition protein to trigger innate immune response in insects. Proc. Natl. Acad. Sci. USA.

[4] Basbous N., Coste F., Leone P., Vincentelli R., Royet J., Kellenberger C., Roussel A. (2011). The Drosophila peptidoglycan-recognition protein LF interacts with peptidoglycan-recognition protein LC to downregulate the Imd pathway. EMBO Rep..

[5] Steiner H. (2004). Peptidoglycan recognition proteins: On and off switches for innate immunity. Immunol. Rev..

[6] Watson F.L., Puttmann-Holgado R., Thomas F., Lamar D.L., Hughes M., Kondo M., Rebel V.I., Schmucker D. (2005). Extensive diversity of Ig-superfamily proteins in the immune system of insects. Science.

[7] Christophides G.K., Vlachou D., Kafatos F.C. (2004). Comparative and functional genomics of the innate immune system in the malaria vector Anopheles gambiae. Immunol. Rev..

[8] Bi J., Ning M., Li J., Zhang P., Wang L., Xu S., Zhong Y., Wang Z., Song Q., Li B. (2020). A C-type lectin with dual-CRD from Tribolium castaneum is induced in response to bacterial challenge. Pest. Manag. Sci..

[9] Royet J., Dziarski R. (2007). Peptidoglycan recognition proteins: Pleiotropic sensors and effectors of antimicrobial defences. Nat. Rev. Microbiol..

[10] Chaput C., Boneca I.G. (2007). Peptidoglycan detection by mammals and flies. Microbes Infect..

[11] Mellroth P., Karlsson J., Steiner H. (2003). A scavenger function for a Drosophila peptidoglycan recognition protein. J. Biol. Chem..

[12] Guan R.J., Mariuzza R.A. (2007). Peptidoglycan recognition proteins of the innate immune system. Trends Microbiol..

[13] Yoshida H., Kinoshita K., Ashida M. (1996). Purification of a peptidoglycan recognition protein from hemolymph of the silkworm, Bombyx mori. J. Biol. Chem..

[14] Werner T., Liu G., Kang D., Ekengren S., Steiner H., Hultmark D. (2000). A family of peptidoglycan recognition proteins in the fruit fly Drosophila melanogaster. Proc. Natl. Acad. Sci. USA.

[15] Christophides G.K., Zdobnov E., Barillas-Mury C., Birney E., Blandin S., Blass C., Brey P.T., Collins F.H., Danielli A., Dimopoulos G. (2002). Immunity-related genes and gene families in Anopheles gambiae. Science.

[16] Tanaka H., Ishibashi J., Fujita K., Nakajima Y., Sagisaka A., Tomimoto K., Suzuki N., Yoshiyama M., Kaneko Y., Iwasaki T. (2008). A genome-wide analysis of genes and gene families involved in innate immunity of Bombyx mori. Insect Biochem. Mol. Biol..

[17] Zhang X., He Y., Cao X., Gunaratna R.T., Chen Y.R., Blissard G., Kanost M.R., Jiang H. (2015). Phylogenetic analysis and expression profiling of the pattern recognition receptors: Insights into molecular recognition of invading pathogens in Manduca sexta. Insect Biochem. Mol. Biol..

[18] Koyama H., Kato D., Minakuchi C., Tanaka T., Yokoi K., Miura K. (2015). Peptidoglycan recognition protein genes and their roles in the innate immune pathways of the red flour beetle, Tribolium castaneum. J. Invertebr. Pathol..

[19] Lemaitre B., Hoffmann J. (2007). The host defense of Drosophila melanogaster. Annu. Rev. Immunol..

[20] Bischoff V., Vignal C., Duvic B., Boneca I.G., Hoffmann J.A., Royet J. (2006). Downregulation of the Drosophila immune response by peptidoglycan-recognition proteins SC1 and SC2. PLos Pathog..

[21] Zaidman-Remy A., Herve M., Poidevin M., Pili-Floury S., Kim M.S., Blanot D., Oh B.H., Ueda R., Mengin-Lecreulx D., Lemaitre B. (2006). The Drosophila amidase PGRP-LB modulates the immune response to bacterial infection. Immunity.

[22] Royet J., Gupta D., Dziarski R. (2011). Peptidoglycan recognition proteins: Modulators of the microbiome and inflammation. Nat. Rev. Immunol..

[23] Kurata S. (2014). Peptidoglycan recognition proteins in Drosophila immunity. Dev. Comp. Immunol.

[24] Chang M.M., Wang Y.H., Yang Q.T., Wang X.L., Wang M., Raikhel A.S., Zou Z. (2020). Regulation of antimicrobial peptides by juvenile hormone and its receptor, Methoprene-tolerant, in the mosquito Aedes aegypti. Insect Biochem. Mol. Biol..

[25] Bischoff V., Vignal C., Boneca I.G., Michel T., Hoffmann J.A., Royet J. (2004). Function of the drosophila pattern-recognition receptor PGRP-SD in the detection of Gram-positive bacteria. Nat. Immunol..

[26] Zhang Z., Kong J., De Mandal S., Li S., Zheng Z., Jin F., Xu X. (2020). An immune-responsive PGRP-S1 regulates the expression of antibacterial peptide genes in diamondback moth, *Plutella xylostella* (L.). Int. J. Biol. Macromol..

[27] Schmidt R.L., Trejo T.R., Plummer T.B., Platt J.L., Tang A.H. (2008). Infection-induced proteolysis of PGRP-LC controls the IMD activation and melanization cascades in Drosophila. FASEB J..

[28] Sumathipala N., Jiang H. (2010). Involvement of Manduca sexta peptidoglycan recognition protein-1 in the recognition of bacteria and activation of prophenoloxidase system. Insect Biochem. Mol. Biol..

[29] Park J.W., Je B.R., Piao S., Inamura S., Fujimoto Y., Fukase K., Kusumoto S., Soderhall K., Ha N.C., Lee B.L. (2006). A synthetic peptidoglycan fragment as a competitive inhibitor of the melanization cascade. J. Biol. Chem..

[30] Wang Q., Ren M., Liu X., Xia H., Chen K. (2020). Identification and characterization of novel short-type BmPGRP-S4 from the silkworm, Bombyx mori, involved in innate immunity. Zeitschrift für Naturforschung C.

[31] Mellroth P., Steiner H. (2006). PGRP-SB1: An N-acetylmuramoyl L-alanine amidase with antibacterial activity. Biochem. Biophys. Res. Commun..

[32] Chen K., Liu C., He Y., Jiang H., Lu Z. (2014). A short-type peptidoglycan recognition protein from the silkworm: Expression, characterization and involvement in the prophenoloxidase activation pathway. Dev. Comp. Immunol..

[33] Afidchao M.M., Musters C.J., de Snoo G.R. (2013). Asian corn borer (ACB) and non-ACB pests in GM corn (*Zea mays.* L.) in the Philippines. Pest. Manag. Sci..

[34] Liu Y., Shen D., Zhou F., Wang G., An C. (2014). Identification of immunity-related genes in Ostrinia furnacalis against entomopathogenic fungi by RNA-seq analysis. PLoS ONE.

[35] Dziarski R., Gupta D. (2006). The peptidoglycan recognition proteins (PGRPs). Genome Biol..

[36] Kim M.S., Byun M.J., Oh B.H. (2003). Crystal structure of peptidoglycan recognition protein LB from Drosophila melanogaster. Nat. Immunol..

[37] Yang D., Su Z., Qiao C., Zhang Z., Wang J., Li F., Liu X. (2013). Identification and characterization of two peptidoglycan recognition proteins with zinc-dependent antibacterial activity from the cotton bollworm, Helicoverpa armigera. Dev. Comp. Immunol..

[38] Yang P.J., Zhan M.Y., Ye C., Yu X.Q., Rao X.J. (2017). Molecular cloning and characterization of a short peptidoglycan recognition protein from silkworm Bombyx mori. Insect Mol. Biol..

[39] Yao F., Li Z., Zhang Y., Zhang S. (2012). A novel short peptidoglycan recognition protein in amphioxus: Identification, expression and bioactivity. Dev. Comp. Immunol..

[40] Swaminathan C.P., Brown P.H., Roychowdhury A., Wang Q., Guan R.J., Silverman N., Goldman W.E., Boons G.J., Mariuzza R.A. (2006). Dual strategies for peptidoglycan discrimination by peptidoglycan recognition proteins (PGRPs). Proc. Natl. Acad. Sci. USA.

[41] Guan R., Roychowdhury A., Ember B., Kumar S., Boons G., Mariuzza R.A. (2004). Structural basis for peptidoglycan binding by peptidoglycan recognition proteins. Proc. Natl. Acad. Sci. USA.

[42] Hu Y., Cao X., Li X., Wang Y., Boons G., Deng J., Jiang H. (2019). The three-dimensional structure and recognition mechanism of Manduca sexta peptidoglycan recognition protein-1. Insect Biochem. Mol..

[43] Lim J.H., Kim M.S., Kim H.E., Yano T., Oshima Y., Aggarwal K., Goldman W.E., Silverman N., Kurata S., Oh B.H. (2006). Structural basis for preferential recognition of diaminopimelic acid-type peptidoglycan by a subset of peptidoglycan recognition proteins. J. Biol. Chem..

[44] Leone P., Bischoff V., Kellenberger C., Hetru C., Royet J., Roussel A. (2008). Crystal structure of Drosophila PGRP-SD suggests binding to DAP-type but not lysine-type peptidoglycan. Mol. Immunol..

[45] Garver L.S., Wu J., Wu L.P. (2006). The peptidoglycan recognition protein PGRP-SC1a is essential for Toll signaling and phagocytosis of Staphylococcus aureus in Drosophila. Proc. Natl. Acad. Sci. USA.

[46] Charroux B., Rival T., Narbonne-Reveau K., Royet J. (2009). Bacterial detection by Drosophila peptidoglycan recognition proteins. Microbes Infect..

[47] Zhao S., Wang X., Cai S., Zhang S., Luo H., Wu C., Zhang R., Zhang J. (2018). A novel peptidoglycan recognition protein involved in the prophenoloxidase activation system and antimicrobial peptide production in Antheraea pernyi. Dev. Comp. Immunol..

[48] An C., Budd A., Kanost M.R., Michel K. (2011). Characterization of a regulatory unit that controls melanization and affects longevity of mosquitoes. Cell. Mol. Life Sci..

[49] Chu Y., Zhou F., Liu Y., Hong F., Wang G., An C. (2015). Ostrinia furnacalis serpin-3 regulates melanization cascade by inhibiting a prophenoloxidase-activating protease. Insect Biochem. Mol. Biol..

