# Supplementary figures and images for: A Short-Type Peptidoglycan Recognition Protein 1 (PGRP1) Is Involved in the Immune Response in Asian Corn Borer, Ostrinia furnacalis (Guenée)
Source: Int J Mol Sci. 2021 Jul 30;22(15):8198. doi: 10.3390/ijms22158198 (PMC8347126; doi:10.3390/ijms22158198)

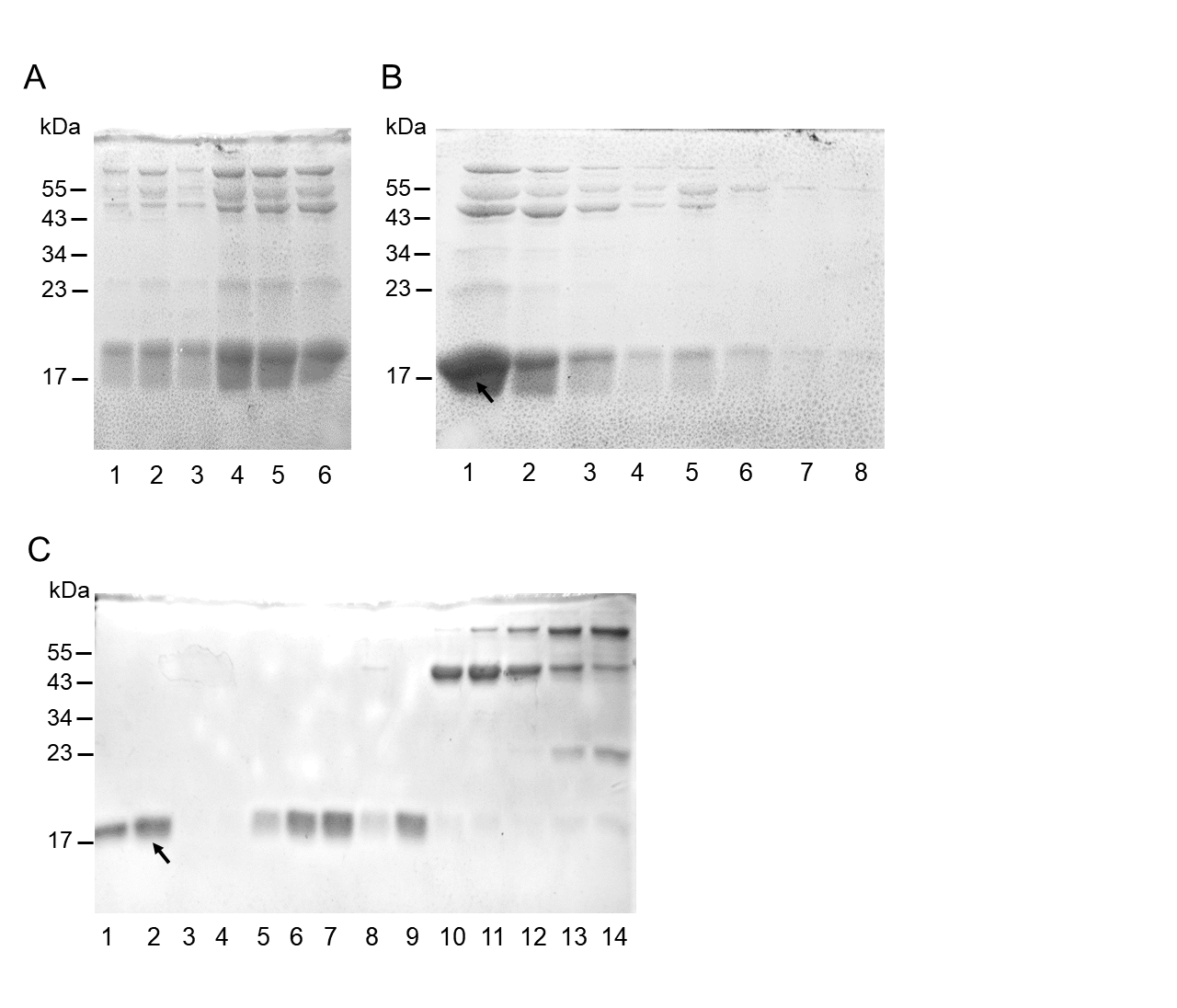

Supplement: Supplementary file 1 [file ijms-22-08198-s001.zip › ijms-1245138-SI/FigS1-purification.tif]

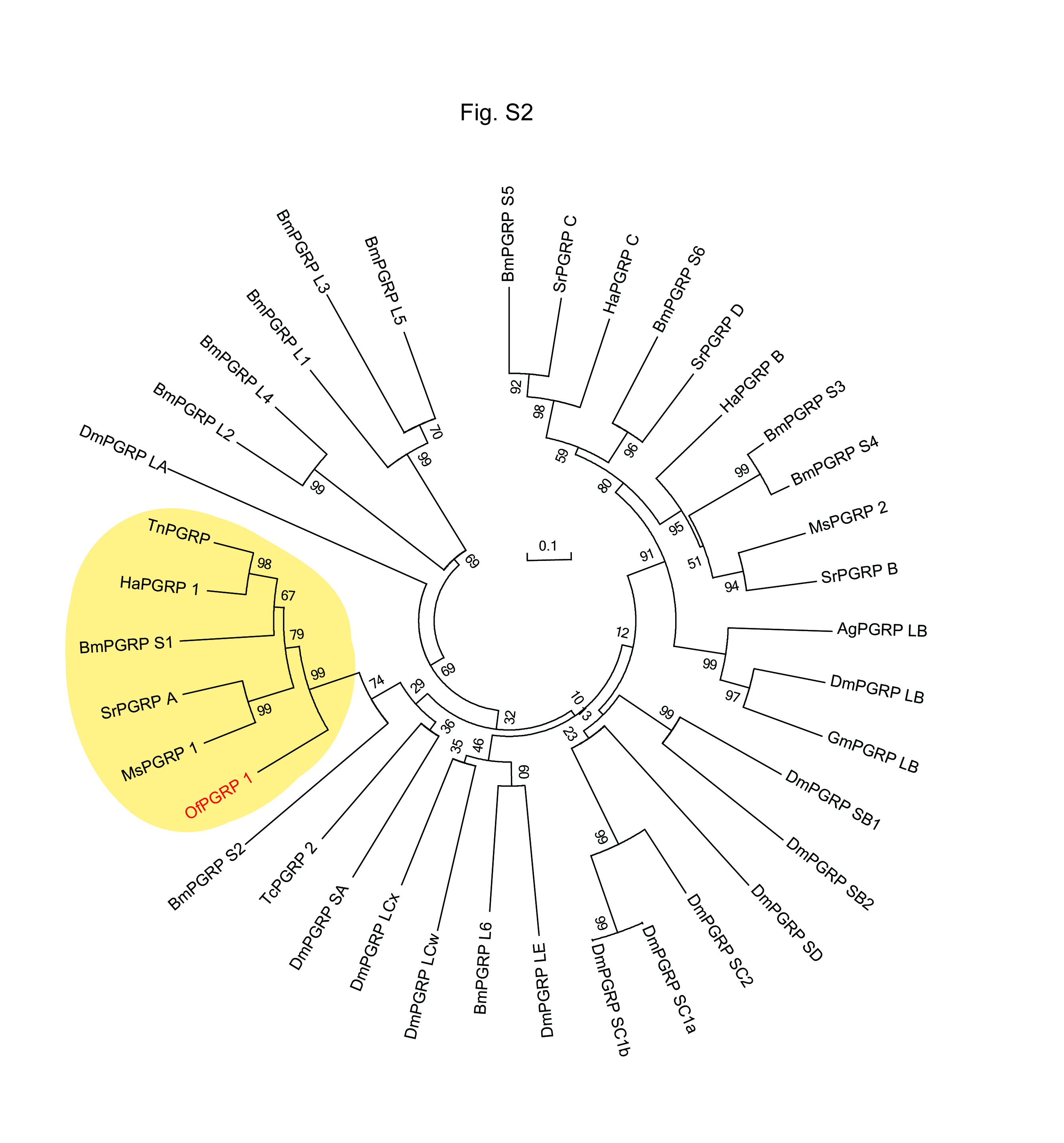

Supplement: Supplementary file 1 [file ijms-22-08198-s001.zip › ijms-1245138-SI/FigS2-Phylogenetic tree.tif]

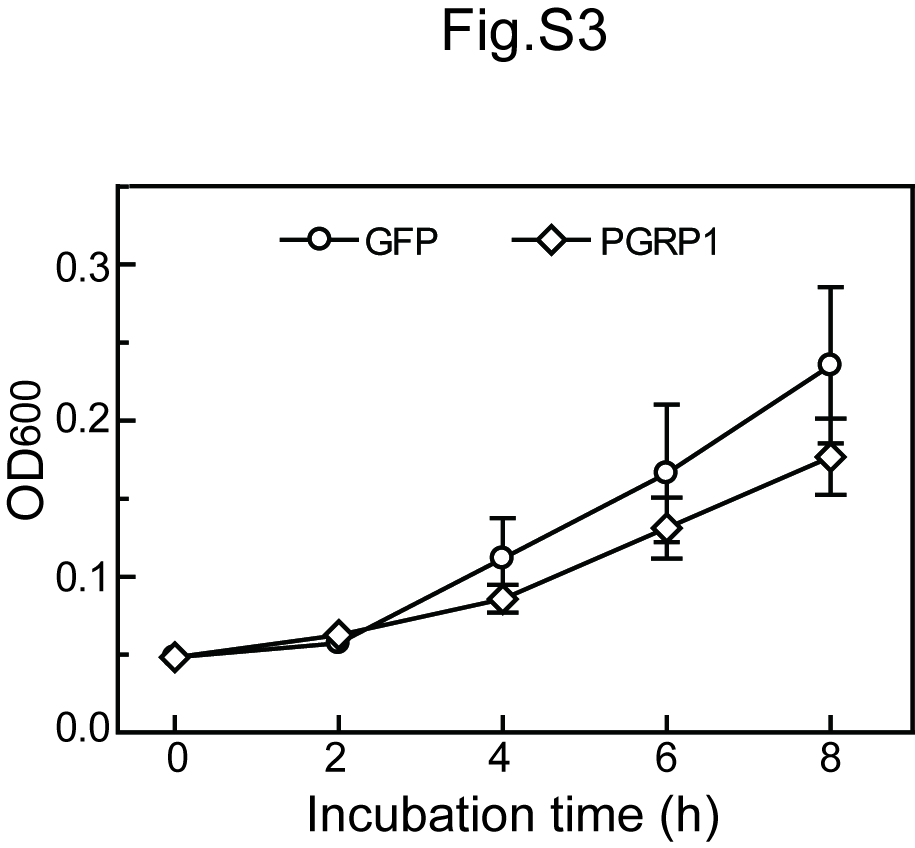

Supplement: Supplementary file 1 [file ijms-22-08198-s001.zip › ijms-1245138-SI/FigS3-No antibactrial activity.tif]

Purification of recombinant protein (Fig. 3)

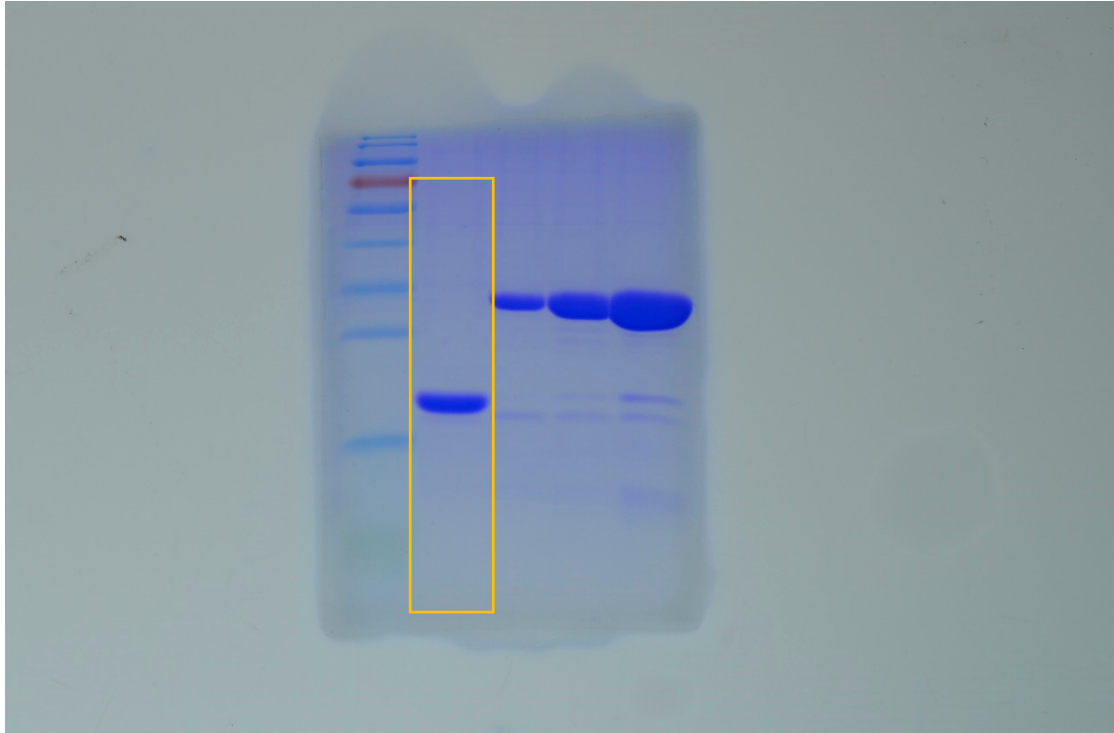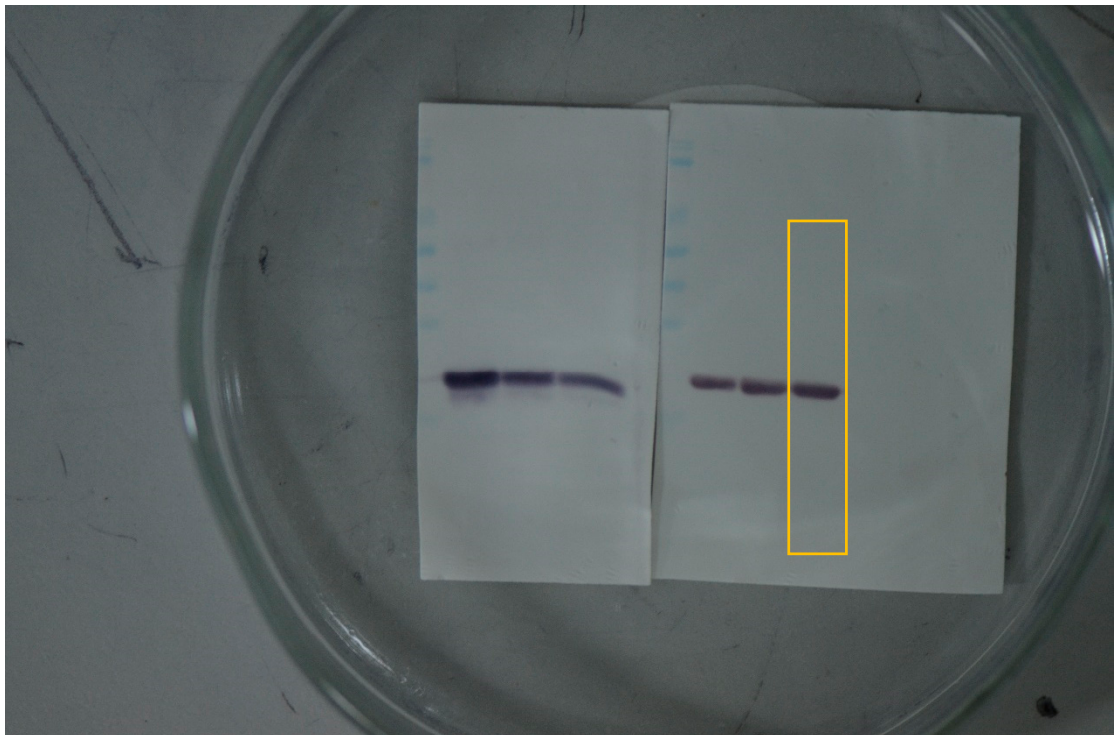

Binding assay (Fig. 4)

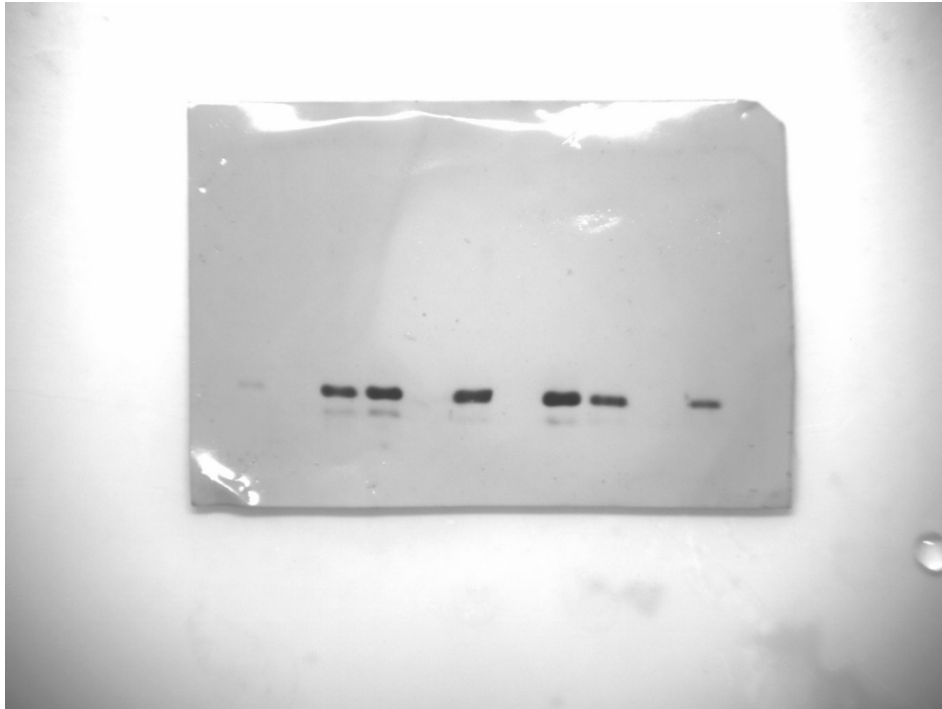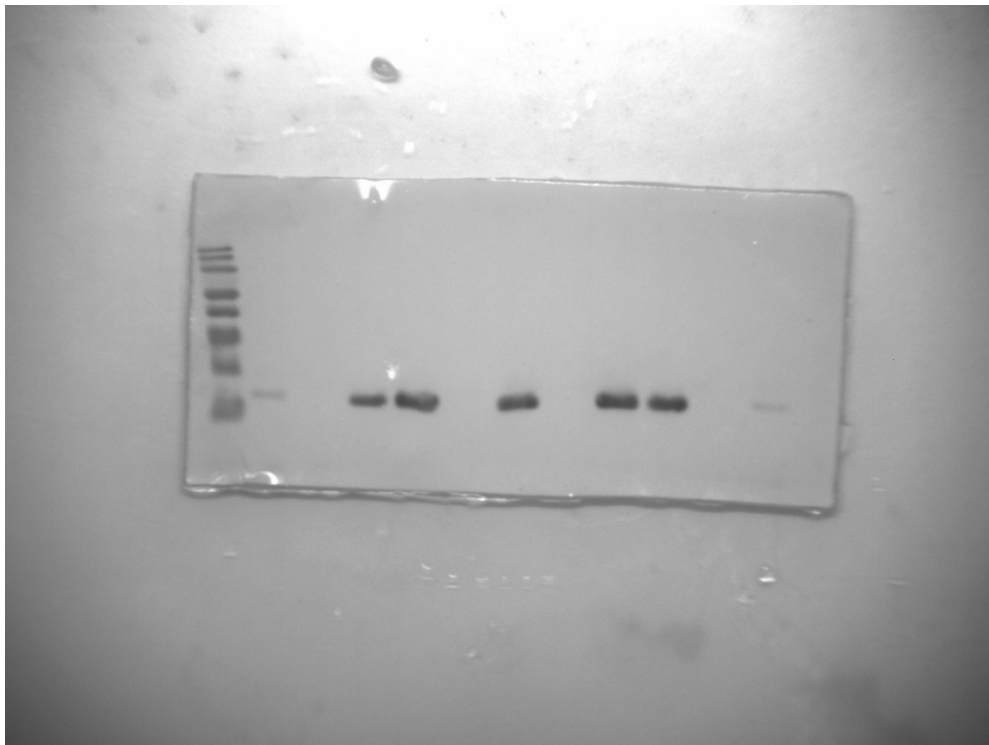

Fig S1

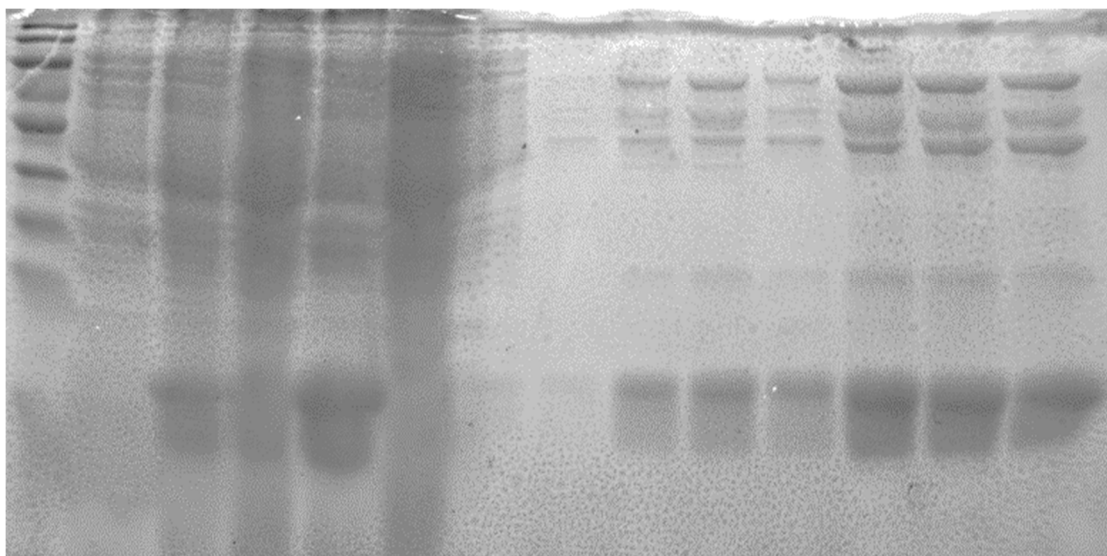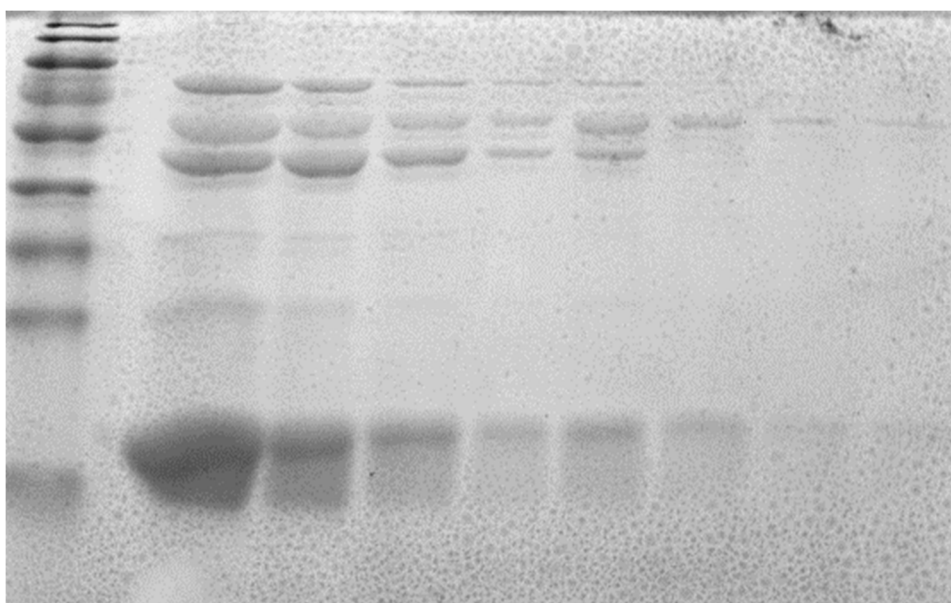

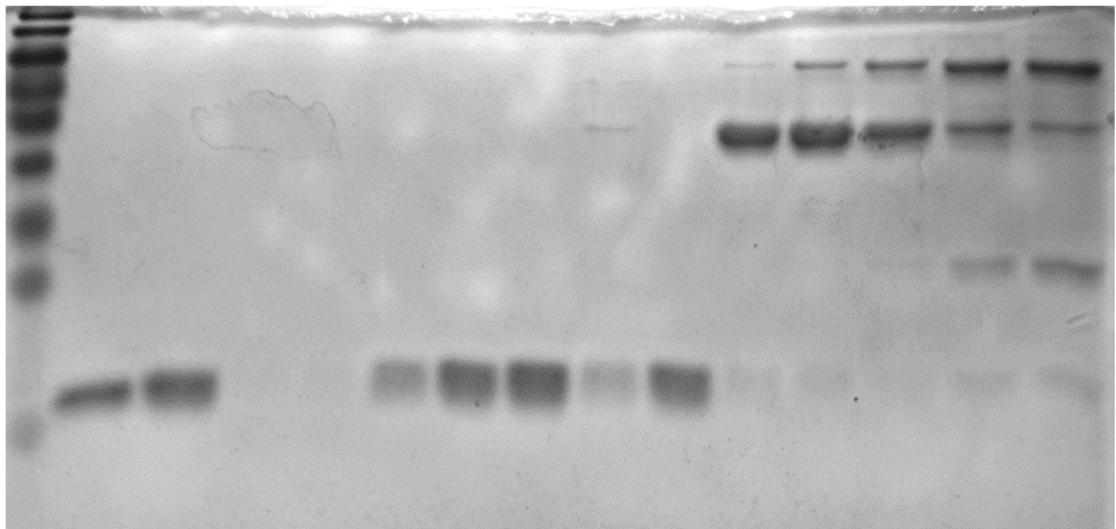

Supplement: Supplementary file 1 [file ijms-22-08198-s001.zip › ijms-1245138-SI/Original Images_NEW.pdf]

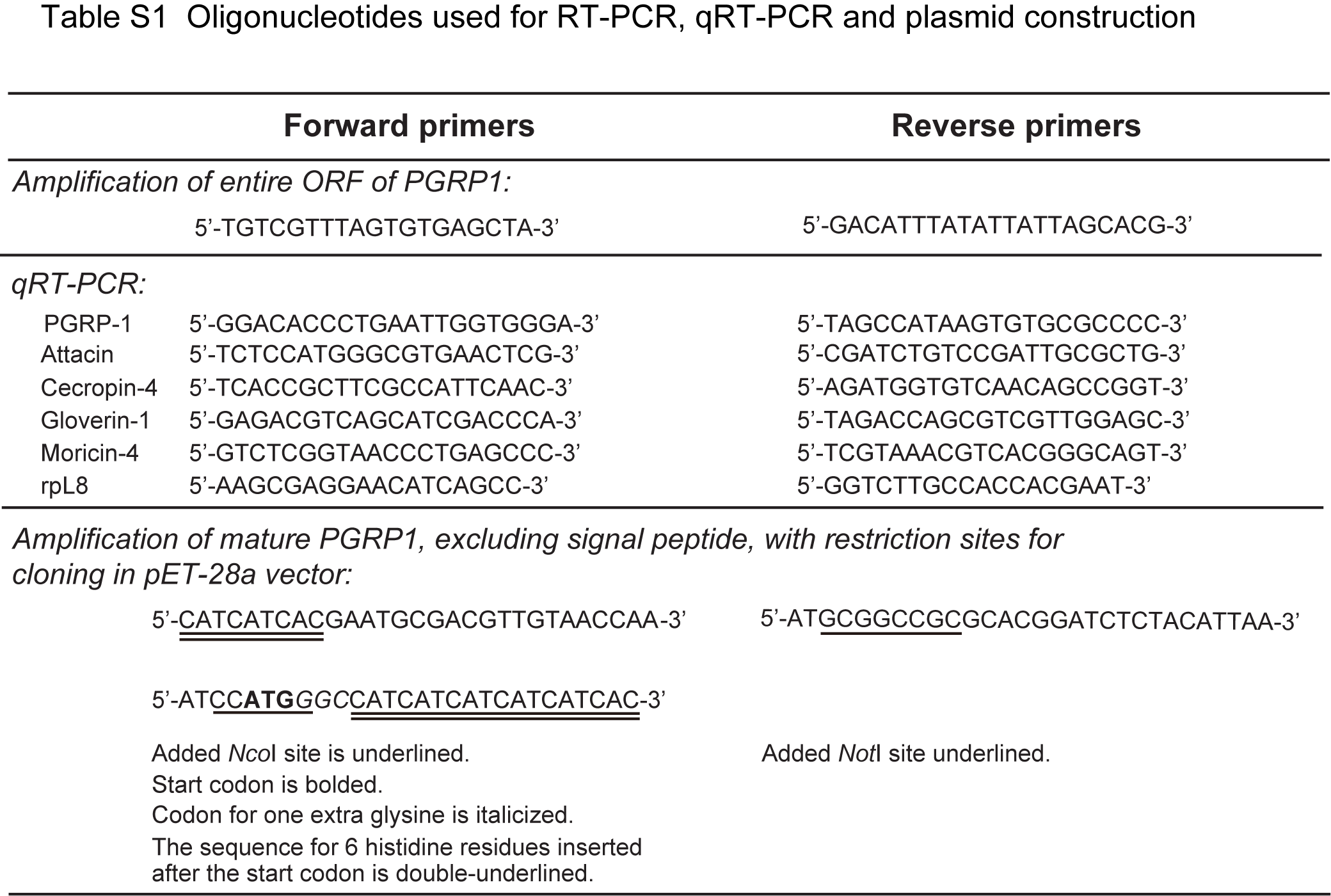

Supplement: Supplementary file 1 [file ijms-22-08198-s001.zip › ijms-1245138-SI/Table-S1-PGRP1 Primer sequences_NEW.tif]
